# Electroacupuncture Pretreatment at GB20 Exerts Antinociceptive Effects via Peripheral and Central Serotonin Mechanism in Conscious Migraine Rats

**DOI:** 10.1155/2016/1846296

**Published:** 2016-10-24

**Authors:** Lu Liu, Pei Pei, Luo-Peng Zhao, Zheng-Yang Qu, Yu-Pu Zhu, Lin-Peng Wang

**Affiliations:** ^1^Acupuncture and Moxibustion Department, Beijing Hospital of Traditional Chinese Medicine, Capital Medical University, No. 23 Meishuguanhou Street, Dongcheng District, Beijing 100010, China; ^2^Neurology Department, Anhui General Hospital of Traditional Chinese Medicine, Anhui University of Chinese Medicine, No. 117 Meishan Street, Anhui 230031, China

## Abstract

*Background*. While electroacupuncture (EA) pretreatment in migraine has been found to attenuate pain and frequencies of attacks, the underlying mechanism of its antinociceptive effect remains poorly understood. Emerging evidence suggests that the serotonin system may be involved in migraine pathophysiology.* Method*. Forty male Sprague-Dawley rats were randomly assigned to Control, Model, EA, and sham acupuncture (SA) groups. HomeCageScan was used to measure the effects on spontaneous nociceptive behaviors. Radioimmunoassay and high-performance liquid chromatography were used to evaluate the expression of 5-hydroxytryptamine (HT) in the plasma and three-key structure of the descending pain modulatory system.* Results*. Our study showed that EA pretreatment could produce a significant reduction in resting, freezing, and grooming behavior and a significant increase in exploration behavior. Furthermore, we found that the level of 5-HT in plasma was significantly increased, and it was significantly decreased in the descending pain modulatory system in Model group. The aforementioned results were significantly reversed in EA group; that is, the level of 5-HT was increased in the rostroventromedial medulla (RVM) and trigeminal nucleus caudalis (TNC) region and decreased in the plasma.* Conclusion*. EA pretreatment exerts antinociceptive effects in a rat model of recurrent migraine, possibly via modulation of the serotonin system.

## 1. Introduction

Migraine is a common and complex brain disorder with significant morbidity, for example, autonomic, cognitive, emotional, and motor disturbance [1]. Apparently, the pathophysiology of migraine consists of alternation of brain excitability, intracranial arterial dilation, recurrent, activation, and sensitization of the trigeminovascular pathway that lead to structural and functional changes in genetically susceptible individual [2–4]; it affects 15% of the population and is characterized by recurrent attacks of severe, often unilateral headaches [5]. Adults aged 25 to 55 years usually suffer from this disease during their productive phase, and migraines cause a lower work productivity and quality of life [6]. Currently, pharmacotherapies, including nonsteroidal anti-inflammatory drugs (NSAIDs) and 5-HT1B/1D receptor agonists, are available for treating acute headaches [7], whereas tricyclics, *β*-blockers, anticonvulsants, and calcium channel blockers are available for preventing headaches. However, there is no “cure” drug for migraine, and many patients have reported intolerance to these drugs because of the side effects [8], which prompts a search for complementary therapies that could be valuable, such as acupuncture [9]. In clinic, numerous randomized controlled trials have demonstrated that acupuncture therapy in migraine can reduce frequencies of attacks for prophylactic intervention with few side effects [10, 11], and it can instantly attenuate pain in the acute phase [12, 13]. However, the underlying mechanism of its antinociceptive effect in migraine remains to be elucidated.

The peripheral and central serotonin system has been shown to play a vital role in migraine pathophysiology via its effects on trigeminovascular nociceptive information transmission and central sensitization [14]. In the peripheral nervous system, there is a consistent increase in plasma serotonin (5-hydroxytryptamine, 5-HT) release concomitant with a decrease in 5-hydroxyindoleacetic acid (HIAA) content during migraine attacks [15, 16]. Conversely, low plasma 5-HT and high 5-HIAA levels have been found between migraine attacks [17]. In the central nervous system (CNS), the serotonergic system from the brainstem rostroventromedial medulla (RVM) region, including the nucleus raphe magnus (NRM) and nucleus reticularis paragigantocellularis (NpGC) nuclei, has descending projections to the trigeminal nucleus caudalis (TNC) and widespread projections to the forebrain through the periaqueductal gray (PAG) [18]. The 5-HT concentration in the CNS can modulate nociceptive processing in either a facilitatory or inhibitory manner. In principle, a high concentration of 5-HT is inhibitory, whereas a low concentration of 5-HT is excitatory [14].

In this study, we hypothesized that acupuncture pretreatment would exert antinociceptive effects in recurrent migraine via the peripheral and central serotonin system. To test this hypothesis, we chose a rat model of recurrent migraine induced by repeated electrical stimulation of the dura surrounding the superior sagittal sinus (SSS), which is an established paradigm of migraine-associated pain [19, 20]. We evaluated the antinociceptive effect of EA on the migraine-like behaviors, such as change in rest, freezing, grooming, and exploration behaviors. In addition, we assessed the 5-HT level in the peripheral plasma and central regions of the descending pain modulatory pathway to determine the underlying mechanism of the EA-induced antinociceptive effect.

## 2. Materials and Methods

### 2.1. Ethical Concerns

All procedures and experimental protocols were approved by the Beijing Institutional Review Board for Animal Experiments (Care and Use Committee of Capital Medical University, Beijing, ethical approval number AEEI 2015-075). The study was performed in accordance with the International Association for the Study of Pain guidelines and Guideline for the Care and Use of Laboratory Animals (State Council of China, 2013). All efforts were made to minimize the animals suffering and reduce the number of animals used.

### 2.2. Sample Size Calculation and Statistical Analysis

The number of rats used in each group was based on the power calculation of previous studies [21, 22]. To determine the sample size requirement, a power calculation for a two-tailed test was performed based on our behavioral outcome measure of grooming (s/h). Based on previous study, we estimated that 10 animals per group would be required to detect 899.8 s/h in EA group, assuming a standard deviation (SD) of 63.37, with an *α* level of 0.05 and 90% power. Based on principles of safeguard animal welfare, we aimed to test the minimal number of animals required for upholding statistical power (10 animals per group) and minimized animal suffering wherever possible.

Data are expressed as mean ± SD. Data analysis was performed by using SPSS V.12.0 (SPSS Inc., Chicago, IL, USA). Parameters for resting, freezing, grooming, and exploration behaviors were analyzed by two-way repeated-measures ANOVA followed by a post hoc least significant difference multiple comparison tests. One-way ANOVA was conducted to compare the results of four behavioral parameters at different time points, RIA assay, and HPLC analysis followed by a post hoc least significant difference multiple comparison tests. A probability level of *p* < 0.05 was set as the threshold of statistical significance.

### 2.3. Experimental Animals

Pathogen-free adult male Sprague-Dawley rats (200–250 g) were purchased from Vital River Laboratories. The rats were housed in temperature (23°C) and light (12 : 12 light/dark cycle) controlled rooms, and standard rodent chow and water were provided ad libitum. After one week of habituation, the rats underwent surgery to establish the migraine model.

### 2.4. Surgical Procedures

#### 2.4.1. Exposure of the Dura Mater

The rats were anesthetized with 60 mg/kg intraperitoneal 2% sodium pentobarbital (Sigma-Aldrich) as described by previous studies [23–25]. Subsequently, each rat was placed in a stereotactic frame. During surgery, sterile ophthalmic ointment was used to protect the rat's eyes. The scalp, which covers the dorsal surface of the skull, was incised, and the connective tissue and muscle were removed, exposing the parietal bone. Two cranial windows (4 mm anterior and 6 mm posterior to the bregma on the midline suture, each 1 mm in diameter) for electrical stimulation were carefully drilled into the parietal bone, and the skull was opened to expose the dura mater adjacent to the SSS [26]. Firstly, the outer layer of the skull was thinned by using a dental drill (78001, RWD Life Science Co., Ltd., Shenzhen, China) and irrigated with a constant application of ice-cold sterile saline to prevent heating. Secondly, the inner layer of the skull was removed by using a curette to expose the intact dura.

#### 2.4.2. Electrode Fixation

A pair of tailored electrode fixtures (Beijing JiAnDeEr Ltd., China), each composed of a bipolar electrode (length 1.9–2.1 mm, diameter 0.8 mm), a plastic holder with two screw holes, and an obturator cap, were inserted into the cranial windows so that they touched the dural surface surrounding the SSS. Then two stainless steel screws (M1.4 × 2.8 mm) were placed into the screw holes as anchors for the dental cement (Shanghai New Century Dental Materials Co, Ltd., Shanghai, China), which was used to stabilize the electrode fixtures. Finally, the obturator cap was placed over the external stimuli line interface to prevent clogging. All surgeries were performed under direct visual control by using an operating microscope. Postoperatively, the rats' breathing and movements were observed until they awoke and walked freely in a clean, plastic cage with a heat blanket. Penicillin (0.04 million IU/100 g, Harbin Pharmaceutical Group Co., Ltd., China) was applied to the lesion to prevent infection for 3 days after the surgery. All the rats were housed separately at a constant temperature and a 12-hour light-dark cycle with unrestricted access to food and water, to recover for 7 days before experiments began.

### 2.5. Experimental Design

**Figure 1:**
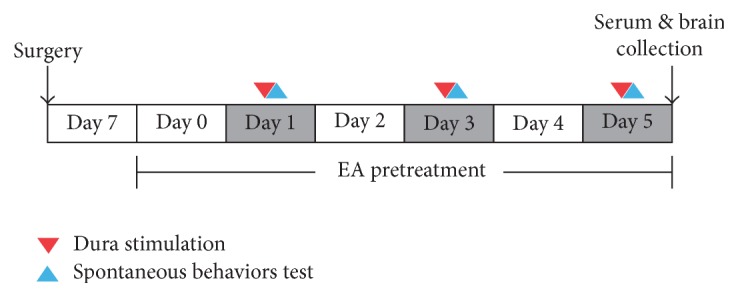
Experimental protocol diagram. One week after surgery, rats received dura electrical stimulation every 2 days from day 1 to day 5 (days 1, 3, and 5). EA pretreatment was performed every day on both dura stimulation period (before dura electrical stimulation) and blank period (days 0, 2, and 4) in EA group. Spontaneous homecage behaviors recording by HomeCageScan was assessed immediately after dura electrical stimulation (days 1, 3, and 5). Plasma 5-HT immunoreactivity and central 5-HT HPLC determination were performed on day 5.

After recovering from the surgery, 40 animals were randomly divided into four groups: an EA group, which received EA at GB20 (*Feng chi*) following dural electrical stimulation; a Sham acupuncture (SA) group, which received acupuncture (without EA and manual manipulation) at a distant nonacupuncture point following electrical stimulation of the dura; a Model group, which received electrical dural stimulation without EA; and a Control group, which received neither electrical dural stimulation nor EA (only electrode implantation). The EA, SA, and Model groups received repeated dural electrical stimulation using a stimulator (YC-2 stimulator, Chengdu Instrument, Co.) every other day for 3 sessions (days 1, 3, and 5). Before electrical stimulation, the EA group received EA at GB20 once daily from day 0 to day 5 for 6 sessions. The experimental protocol diagram was depicted in Figure 1.

#### 2.5.1. EA at GB20

**Figure 2:**
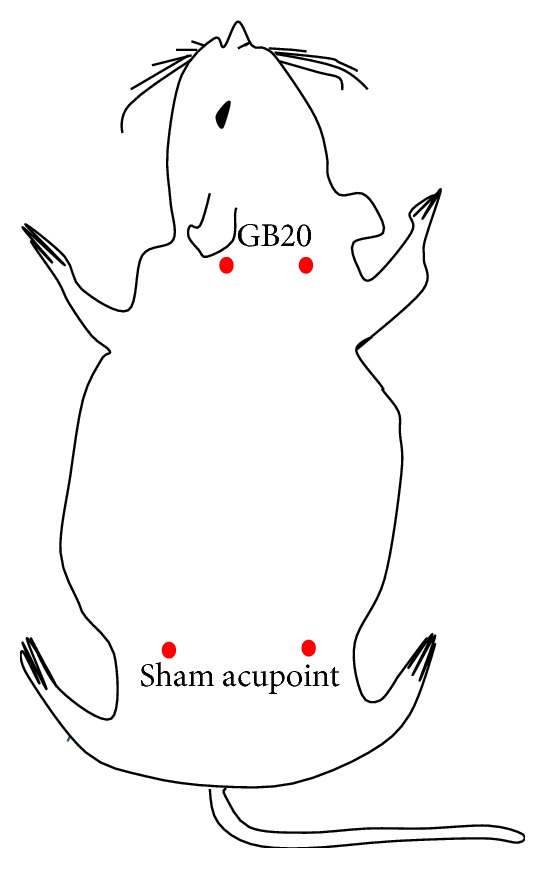
Rat schematic showing the location of the acupuncture points used in the study. GB20 stands for “*Feng chi,*” which is located at 3 mm away from the center of a line joining two ears. Sham acupuncture point is located at about 10 mm above iliac crest.

Before dural electrical stimulation, rats in EA and SA group received EA/SA pretreatment from days 0 to 5, for 6 sessions. The rats were placed into a tailored apparatus that exposed their heads and necks sufficiently. GB20 is a commonly used acupuncture point for treating migraines [11]. In rats, the point is anatomically similar to that in humans, as it is located 3 mm lateral to the center of a line joining the two ears at the back of the head. In the EA group, two stainless steel acupuncture needles (diameter 0.25 mm, length 25 mm; Suzhou Medical Appliance Factory, Suzhou, China) were inserted bilaterally at GB20 to a depth of 2-3 mm in the direction of the opposite eye. Next, the needle handle was connected to an electrical needle stimulator (Han's acupuncture point nerve stimulator HNAS-200, Nanjing, China) between 08:00 a.m. and 10:00 a.m. for 15 min/days. The EA parameters were as follows: 2/15 Hz frequency (amplitude-modulated wave) and 0.5–1.0 mA intensity (depending on the reaction of the rat). Correct placement of the needles at the acupoints was confirmed by observing slight repetitive auricle twitches during EA stimulation [27]. In the SA group, needles were placed bilaterally at distant nonacupuncture points (about 10 mm above iliac crest) at a depth of 2-3 mm [28], but no stimulation was administered. The animals in the Control and Model groups were placed into the apparatus in a similar manner for 15 min, but no acupuncture was performed. All rats were conscious during EA implementation. The aforementioned acupuncture points were shown in Figure 2.

#### 2.5.2. Repeated Electrical Stimulations on the Dura

Before electrical stimulation was performed, the rats were individually placed into an observation cage (diameter 40 cm, height 17.5 cm) and habituated for 20 min to minimize stress. Then the obturator cap was removed from the electrode fixtures, and an internal delivery electrode tip was inserted, which was connected to the current external output port of an electrical stimulator (YC-2 stimulator, Chengdu Instrument, Co.). On the basis of previous studies [19, 20, 26] and our own optimization of stimulation, effective dural stimuli consisted of monophasic square-wave pulses with a pulse duration of 0.5 ms at an intensity of 1.8–2.0 mA and frequency of 20 Hz, and stimulation was administered to rats in the EA, SA, and Model groups for 15-minute every other day for 3 sessions. The rats in the Control group were also connected to the stimulator, but they received no electrical stimulation.

### 2.6. Data Collection and Analysis

Spontaneous behaviors were monitored by using automated behavioral acquisition and analysis software, HomeCageScan (Clever Systems Inc., Reston, Virginia, USA). Before real/sham electrical dural stimulation, the rats were acclimatized for 20 min in an individual transparent acrylic observation cage with an automatic real-time video surveillance and image analysis system, which was illuminated to mimic daytime conditions. Subsequently, the rats received electrical dural stimulation for 15 min in another cage before being individually placed back into the observation cage for 45 min recording.

The observation cage was cleaned with 10% ethanol between sessions to eliminate traces of odor. The HomeCageScan software used information from the rats' entire body, including the mouth, head, tail, forelimbs, hind limbs, upper/lower back, and abdomen, and sequence data to automatically recognize and analyze rats behaviors in periods of >6 frames (30 frames/s). All the behaviors were verified and calibrated the preceding recording and analysis. During the test, rats were free to access food and water in the transparent observation cage, which contained sawdust bedding. Specific spontaneous nociceptive behaviors have been analyzed previously, and they were often observed in the analysis of experimental migraine models induced by dural nociception [23]. These behaviors are described as follows:

1. Resting behavior: resting the head on flexed forepaws, without rearing or hanging.
2. Freezing behavior: remaining low and stationary and pausing.
3. Grooming behavior: cleaning the body and face by making circular movements with the forepaws.
4. Exploration behavior: walking, hanging, rearing, and sniffing.

### 2.7. Plasma 5-HT Level Analysis

Following the completion of study period, 3 mL of jugular venous blood sample was obtained and aliquoted in an ice-cold tube containing ethylenediaminetetraacetic acid (EDTA) (7.5% 0.072 mL/3 mL blood) and aprotinin (2,700 KIU/3 mL blood). Then the samples were centrifuged at 3,000 rpm for 10 min at 4°C, and the plasma supernatant was aspirated and stored at −80°C until the level of plasma 5-HT was determined. The plasma 5HT concentrations were measured by the specific and sensitive radioimmunoassay (RIA) method. A 3 mL aliquot of freeze-dried plasma was reconstituted, and immunoreactive 5-HT was quantitated using an Iodine [125I] 5-HT Radioimmunoassay Kit (Beijing Purevalley Biotech Co., Ltd., China). The detection limit was 15 pg/mL, interassay coefficient of variation was <12% in the 20–1,200 pg/mL range, and intra-assay coefficient of variation was <7%.

### 2.8. Cerebral 5-HT Level Analysis

High-performance liquid chromatography (HPLC) with electrochemical detector was used to assess the 5-HT level from the cerebral homogenate samples. At the completion of experiment (Day 5), rats were sacrificed and brains were rapidly removed, frozen by using liquid nitrogen, and stored until dissection at −80°C. Dissection of brain areas was performed on a frozen microtome. Bilateral punches of the PAG, RVM, and TNC regions [29] were selected by using a magnifying glass from frontal brain sections (300 *μ*m) with stainless steel cannulae of 1,000 *μ*m inner diameter and pooled. Samples were stored in preweighed, small Eppendorf tubes and stored at −80°C. On the day of the HPLC analysis, after weighing the Eppendorf tube, 1 mg of brain tissue of different regions was collected and homogenized in 0.1 mol/L analytical grade perchloric acid (HCLO_4_) by using an ultrasonic cell crusher. The homogenate was centrifuged at a speed of 12,000 r/min for 15 min at 4°C, and 20 *μ*L supernatant was obtained and directly injected into a Waters 2695 series HPLC (Waters Alliance, 2695, USA) equipped with C18 Column.

Determination conditions of HPLC are as follows: Atlantis C18 Column (2.1 × 150 mm, 3 *μ*m, Waters Atlantis, USA); Electrochemical Detector (Waters Alliance, 2465, USA); Mobile Phase: 50 mM citric acid (C_6_H_8_O_7,_ analytical grade), sodium acetate (C_2_H_3_O_2_Na, analytical grade), buffer solution (pH 3.5, 1.8 mM dibutylamine, 0.3 mM Na_2_EDTA, Sigma), and methanol (CH_3_OH, HPLC grade) (96 : 4, v/v); Column Temperature: 35°C; Detection Voltage: +0.75 V; Flow Rate: 0.35 mL/min.

## 3. Results

### 3.1. Effect of EA Pretreatment on Migraine-Like Spontaneous Behaviors

#### 3.1.1. Resting Behavior

**Figure 3:**
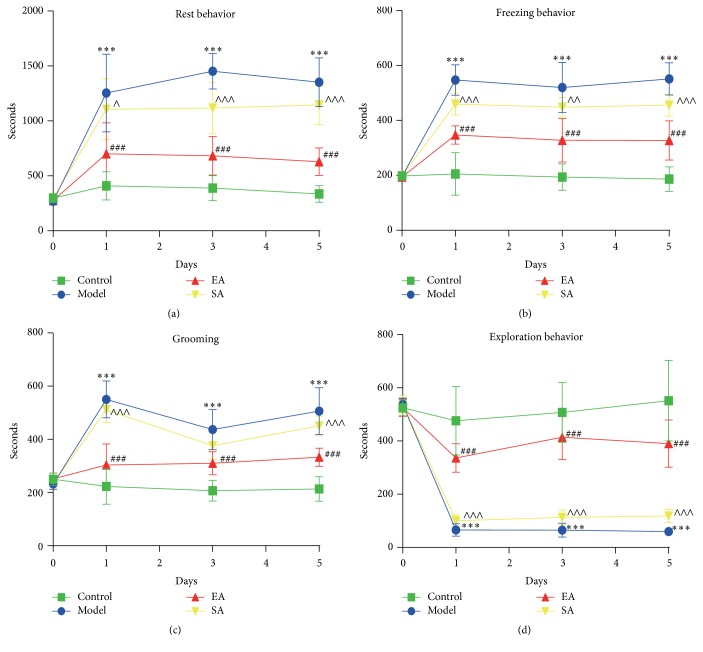
Effect of EA pretreatment on migraine-like spontaneous behavior on days 1, 3, and 5. The figure depicted the average in seconds of (a) resting behavior, (b) freezing behavior, (c) grooming, and (d) exploration behavior during 45 min after dura electrical stimulation. Data are mean ± SD (*n* = 10 per time point per group). ^*∗∗∗*^ *p* < 0.001, Model versus Control. ^###^ *p* < 0.001, EA versus Model. ^∧^ *p* < 0.05, ^∧∧^ *p* < 0.01, and ^∧∧∧^ *p* < 0.001, SA versus EA.

EA treatment significantly decreased the average resting behavior within 45 min after dural stimulation (repeated-measures ANOVA, *p* < 0.0001, Figure 3(a)), suggesting that EA could ameliorate the increased resting behavior induced by dural stimulation. A significant difference in the resting behavior was observed between the EA and SA groups (repeated-measures ANOVA post hoc, *p* < 0.0001, Figure 3(a)). Then we analyzed the average resting behavior among the four different groups on days 1, 3, and 5. A significantly increased resting behavior was found over the three days in the Model group compared to the Control group (day 1: 1254.89 ± 353.29 s versus 408.54 ± 128.17 s, *p* < 0.001; day 3: 1452.39 ± 161.39 s versus 389.51 ± 113.87 s, *p* < 0.001; day 5: 1351.74 ± 222.19 s versus 355.08 ± 76.43 s, *p* < 0.001, resp.; Figure 3(a)). Furthermore, the dural stimulation-induced increase in resting behavior was significantly decreased with EA pretreatment at GB20 on days 1, 3, and 5 in the EA group compared to the model group (day 1: 701.35 ± 282.61 s versus 1254.89 ± 353.29 s, *p* < 0.001; day 3: 683.26 ± 176.32 s versus 1452.39 ± 161.39 s, *p* < 0.001; day 5: 628.56 ± 125.65 s versus 1351.74 ± 222.19 s, *p* < 0.001, resp.; Figure 3(a)). Compared to the EA group, rats in the SA group spent more time resting on days 1, 3, and 5 (*p* = 0.013, *p* < 0.001, and *p* < 0.001, resp.; Figure 3(a)). In contrast, there were no significant differences between the Model and SA groups on days 1 and 5 (*p* = 1.000, *p* = 0.193, resp.).

#### 3.1.2. Freezing Behavior

EA treatment significantly decreased the average freezing behavior within 45 min after dural stimulation (repeated-measures ANOVA, *p* < 0.0001, Figure 3(b)), suggesting that EA could ameliorate the increased freezing behavior induced by dural stimulation. A significant difference in the freezing behavior was observed between the EA and SA groups (repeated-measures ANOVA post hoc, *p* < 0.0001, Figure 3(b)). Then we analyzed the average freezing behavior among the four different groups on days 1, 3, and 5. After the meningeal electrical stimulation, the Model group displayed a significant increase of the freezing behavior on days 1, 3, and 5 compared to the Control group (day 1: 546.77 ± 55.75 s versus 204.71 ± 77.32 s, *p* < 0.001; day 3: 519.46 ± 91.32 s versus 193.73 ± 47.87 s, *p* < 0.001; day 5: 551.01 ± 58.83 s versus 186.58 ± 44.83 s, *p* < 0.001, resp.; Figure 3(b)). In addition, the freezing behavior in EA group reduced significantly more than that in the Model group over the three days (day 1: 346.99 ± 33.11 s versus 546.77 ± 55.75 s, *p* < 0.001; day 3: 327.64 ± 79.67 s versus 519.46 ± 91.32 s, *p* < 0.001; day 5: 326.85 ± 71.42 s versus 551.01 ± 58.83 s, *p* < 0.001, resp.; Figure 3(b)). The rats spent more time engaging in the freezing behavior on days 1, 3, and 5 (*p* < 0.001, *p* < 0.01, and *p* < 0.001, resp.; Figure 3(b)) in the SA group compared to the EA group.

#### 3.1.3. Grooming Behavior

EA treatment significantly decreased the average grooming behavior within 45 min after dural stimulation (repeated-measures ANOVA, *p* < 0.0001, Figure 3(c)), suggesting that EA could ameliorate the increased grooming behavior induced by dural stimulation. A significant difference in the grooming behavior was observed between the EA and SA groups (repeated-measures ANOVA post hoc, *p* < 0.0001, Figure 3(c)). Then we analyzed the average grooming behavior among the four different groups on days 1, 3, and 5. As shown in Figure 3(c), the analysis over the three days showed a significant increase in grooming time in the Model group as compared to the Control group (day 1: 538.85 ± 74.69 s versus 223.19 ± 66.58 s, *p* < 0.001; day 3: 437.25 ± 75.56 s versus 207.21 ± 39.03 s, *p* < 0.001; day 5: 506.14 ± 88.23 s versus 213.87 ± 46.43 s, *p* < 0.001, resp.; Figure 3(c)). Pretreatment with EA at GB20 demonstrated a significant effect in decreasing the dura stimulation-induced increase in grooming behavior in migraine rats (day 1: 303.61 ± 79.52 s versus 538.85 ± 74.69 s, *p* < 0.001; day 3, 310.63 ± 43.82 s versus 437.25 ± 75.56 s, *p* < 0.001; day 5, 332.86 ± 33.97 s versus 506.14 ± 88.23 s, *p* < 0.001, resp.; Figure 3(c)). Compared to the SA group, the EA group spent a significantly decreased amount of time on grooming on days 1 and 5 (*p* < 0.001, *p* < 0.001, resp.; Figure 3(c)), although this decrease was not significant on day 3 (*p* = 0.62; Figure 3(c)). In contrast, there were no significant differences between the Model and SA groups on days 1, 3, and 5 (*p* = 1.000, *p* = 1.000, and *p* = 0.44, resp.).

#### 3.1.4. Exploration Behavior

EA treatment significantly increased the average exploration behavior within 45 min after dural stimulation (repeated-measures ANOVA, *p* < 0.0001, Figure 3(d)), suggesting that EA could enhance the decreased exploration behavior induced by dural stimulation. A significant difference in exploration behavior was observed between the EA and SA groups (repeated-measures ANOVA post hoc, *p* < 0.0001, Figure 3(d)). Then we analyzed the average grooming behavior among the four different groups on days 1, 3, and 5. There was a significant decrease in the time spent engaging in exploration behavior in the Model group compared to the Control group (day 1: 65.49 ± 23.64 s versus 476.34 ± 128.55 s, *p* < 0.001; day 3, 64.88 ± 26.34 s versus 507.61 ± 112.52 s, *p* < 0.001; day 5, 59.93 ± 15.45 s versus 551.78 ± 151.4 s, *p* < 0.001, resp.; Figure 3(d)). Exploration behavior over the three days increased significantly in the EA group compared to the Model group (day 1, 336.37 ± 53.4 s versus 65.49 ± 23.64 s, *p* < 0.001; day 3, 414.28 ± 83.21 s versus 64.88 ± 26.34 s, *p* < 0.001; day 5, 390.32 ± 88.63 s versus 59.93 ± 15.45 s, *p* < 0.001, resp.; Figure 3(d)). The dural stimulation-induced reduction in exploration behavior was significantly increased with EA pretreatment at GB20 on days 1, 3, and 5 compared to the SA group (*p* < 0.001, *p* < 0.001, and *p* < 0.001, resp.; Figure 3(d)).

### 3.2. Effect of EA Pretreatment on 5-HT Level of Peripheral Serum in Conscious Migraine Rats

**Figure 4:**
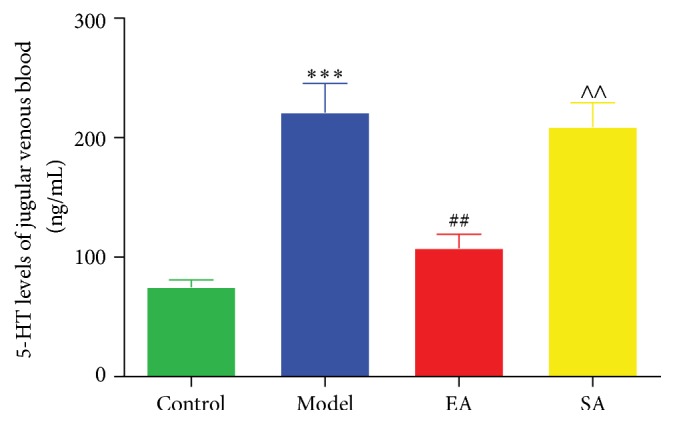
Effect of EA pretreatment on 5-HT level of peripheral serum in conscious migraine rats. 5-HT levels of jugular venous blood samples were determined by radioimmunoassay (RIA) method. Data are mean concentration (ng/mL) ± SD (*n* = 6 for each group). ^*∗∗∗*^ *p* < 0.001, Model versus Control. ^##^ *p* < 0.01, EA versus Model. ^∧∧^ *p* < 0.01, SA versus EA.

Repetitive electrical stimulation on the dura significantly increased the serum levels of 5-HT in the Model group compared to the Control group (221.77 ± 60.52 ng/mL versus 75.18 ± 15.34 ng/mL, *p* < 0.001; Figure 4). The repetitive electrical stimulation-induced serum 5-HT increase in rats was significantly inhibited following EA pretreatment at GB20 (EA group versus Model group: 107.94 ± 28.58 ng/mL versus 221.77 ± 60.52 ng/mL, *p* < 0.01; Figure 4), whereas there was no significant effect on dural stimulation-induced serum 5-HT increase in the SA group compared to the Model group (*p* = 1.00; Figure 4).

### 3.3. Effect of EA Pretreatment on 5-HT Level of Endogenous Pain Modulatory System in Conscious Migraine Rats

**Figure 5:**
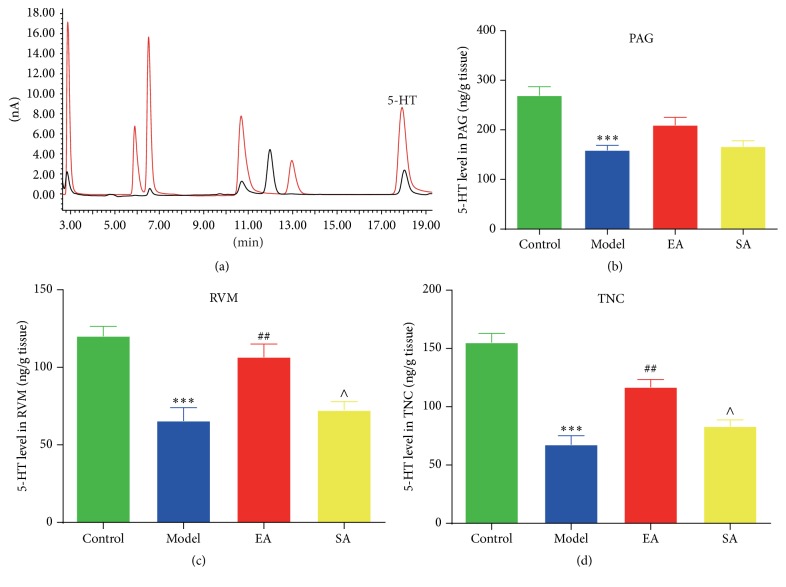
Effect of EA pretreatment on 5-HT level of endogenous pain modulatory system in migraine rats. (a) High-performance liquid chromatography chromatograms of extracted samples. Red curve indicates internal standards; black curve indicates samples. 5-HT was eluted at around 18 min. 5-HT concentrations in the periaqueductal gray (PAG), rostroventromedial medulla (RVM), and trigeminal nucleus caudalis (TNC) were measured by high-performance liquid chromatography (b, c, d). Data are mean concentration (ng/g tissue) ± SD (*n* = 6 for each group). ^*∗∗∗*^ *p* < 0.001, Model versus Control. ^##^ *p* < 0.01, EA versus Model. ^∧^ *p* < 0.05, SA versus EA.

According to the result of the specificity experiment, 5-HT was eluted at around 18 min (Figure 5(a)). Repeated electrical stimulations on the dura significantly inhibited the 5-HT level in the PAG (Model group versus Control group: 158.77 ± 25.04 ng/g tissue versus 269.02 ± 44.35 ng/g tissue, *p* < 0.001; Figure 5(b)), RVM (65.42 ± 21.2 ng/g tissue versus 120.02 ± 15.74 ng/g tissue, *p* < 0.001; Figure 5(c)), and TNC (67.34 ± 19.49 ng/g tissue versus 154.91 ± 19.42 ng/g tissue, *p* < 0.001; Figure 5(d)). The repeated electrical stimulation-induced decreases in the 5-HT level were significantly enhanced with EA pretreatment at GB20 in the RVM (EA group versus Model group: 106.59 ± 20.82 ng/g tissue versus 65.42 ± 21.2 ng/g tissue, *p* < 0.01; Figure 5(c)) and TNC region (116.75 ± 16.24 ng/g tissue versus 67.34 ± 19.49 ng/g tissue, *p* < 0.01; Figure 5(d)), but not in the PAG (209.55 ± 38.6 ng/g tissue versus 158.77 ± 25.04 ng/g tissue, *p* = 0.124; Figure 5(b)). There were no significant differences between the Model and SA groups in terms of the PAG, RVM, and TNC (*p* = 1.000, *p* = 1.000, and *p* = 0.801, resp.).

## 4. Discussion

The present study's finding shows that EA pretreatment at GB20 can ameliorate migraine-like spontaneous behaviors, and EA may exert antinociceptive effects via modulation of the serotonin system in a migraine model.

The behavioral experimentation showed that the repetitive dural electrical stimulations produced an increase in spontaneous nociceptive behaviors, including resting, freezing, and grooming, whereas it decreased the exploration behavior (Figure 3). These behavioral results are in accordance with those of previous studies on clinical symptoms of migraineurs characterized by a reduction in routine physical activity and movement, and an increase in intense hemifacial touching [5, 30]. Moreover, our results suggest that EA pretreatment can exert antinociceptive effects by relieving the migraine-like behaviors. As shown in Figure 3, EA at GB20 produced a decrease in resting, freezing, and grooming behaviors, and an increase in exploration behavior in migraine rats. EA pretreatment decreases the dural electrical stimulations-evoked response without reaching response values in the Control group, and this finding is similar to that demonstrated in migraineurs [11, 31, 32], in which acupuncture is not completely effective but helps to diminish pain during migraine attacks.

Sufficient evidence indicates that EA regulates endogenous opioid peptides (e.g., encephalin and endorphin) and monoamines (e.g., serotonin and norepinephrine) in the central nervous system to exert its antinociception effects [33–35]. However, there is no relevant experimental research of EA for migraine. In the current study, EA pretreatment significantly ameliorated migraine-like spontaneous behaviors. Furthermore, to explore the underlying mechanism of EA-induced antinociceptive effect, we assessed the 5-HT level in the peripheral plasma and central regions of the descending pain modulatory pathway.

Serotonin is implicated in migraine pathophysiology [14, 36, 37]. A low central 5-HT disposition is associated with an increase in the peripheral 5-HT release during a migraine attack, and this is the most convincing change of 5-HT metabolism in migraine [14, 38]. 5-HT interacts with different receptors on trigeminovascular pathway and regulates the nociceptive information. As we know, 5-HT has dual functions, a pronociceptive or antinociceptive effect, depending on which the 5-HT receptor subtype is activated [39].

In the peripheral serotonergic system, 5-HT acts as one of the mediators of neurogenic inflammation, which can be actively released by platelets, immune cells, and mast cells [40]. Peripherally released 5HT around meningeal blood vessels initiates the activation and maintenance of mechanical hypersensitivity of meningeal nociceptors during migraine, which leads to consequent sensitization of central trigeminovascular neurons [41], and evokes thermal hyperalgesia [42]. 5-HT is pronociceptive in the periphery where nociception is often initiated. Our results indicated that repetitive electrical stimulation on the dura significantly increased the serum levels of 5-HT. We also found the downregulation of 5-HT serum after EA pretreatment at GB20 in comparison with migraine rats (Figure 4). In the CNS, changes in serotonin metabolism and processing of the central 5-HT-mediated responses during migraine attacks and interictal periods are well established where migraine is a consequence of a central neurochemical imbalance that involves a low serotonergic disposition [14, 43]. Recent evidence suggests that a low central 5-HT facilitates the activation of the trigeminovascular nociceptive pathway [14]. Our results demonstrated that EA could exert antinociceptive effect via enhancing the concentration of 5-HT in the RVM and TNC regions, but not in PAG (Figure 5). Consistent with our findings, 5-HT upregulation in the central serotonergic system by EA has been reported previously in other pain-related models [44].

By evaluating the expression of 5-HT in the endogenous pain modulatory system and plasma, we found that EA may induce antinociceptive effect by attenuating peripheral 5-HT release and increasing central 5-HT level in the RVM and TNC, not in the PAG. The role of EA in regulating the 5-HT transmission has been reported in many studies [45, 46], which were in agreement with the results of our study. However, we evaluated the serum concentrations and neurotransmitters level of 5-HT in three key structures of the descending pain modulatory system in migraine rats, which is a limitation of this study, as there is no direct evidence that 5-HT receptors are selectively activated or inhibited. In the future, further and more specific studies on the 5-HT receptors are needed. Additional study will also include a group of normal rats to determine that the 5-HT is key neurotransmitter involved in ameliorating migraine attacks.

## 5. Conclusions

Our data demonstrated that the antinociceptive effect of EA in migraine rats occurred via regulation of the peripheral and central serotonin mechanism. This study provides new insight into the mechanism of acupuncture-induced antinociception in migraine.

## Abbreviations

EA: Electroacupuncture
SSS: Superior sagittal sinus
SA: Sham acupuncture
HPLC: High-performance liquid chromatography
5-HT: 5-Hydroxytryptamine
PAG: Periaqueductal gray
RVM: Rostroventromedial medulla
TNC: Trigeminal nucleus caudalis
NSAIDs: Nonsteroidal anti-inflammatory drugs
5-HIAA: 5-Hydroxyindoleacetic acid
CNS: Central nervous system
NRM: Nucleus raphe magnus
NpGC: Nucleus reticularis paragigantocellularis
RIA: Radioimmunoassay.
